# Clinicopathological values of PD-L1 expression in HER2-positive breast cancer

**DOI:** 10.1038/s41598-019-52944-6

**Published:** 2019-11-13

**Authors:** Sasagu Kurozumi, Kenichi Inoue, Hiroshi Matsumoto, Takaaki Fujii, Jun Horiguchi, Tetsunari Oyama, Masafumi Kurosumi, Ken Shirabe

**Affiliations:** 1 Department of General Surgical Science, Gunma University Graduate School of Medicine, Gunma, Japan; 2 Division of Breast Surgery, Saitama Cancer Center, Saitama, Japan; 3 Division of Breast Oncology, Saitama Cancer Center, Saitama, Japan; 4 Department of Breast Surgery, International University of Health and Welfare, Chiba, Japan; 5 Department of Diagnostic Pathology, Gunma University Graduate School of Medicine, Gunma, Japan; 6 Department of Pathology, Saitama Cancer Center, Saitama, Japan

**Keywords:** Predictive markers, Breast cancer, Tumour immunology

## Abstract

Several ongoing clinical trials are investigating the use of immuno-targeting therapy with programmed cell death protein-1 and programmed death-ligand 1 (PD-L1) inhibitors for triple-negative breast cancer. However, the role of PD-L1 expression in HER2-positive breast cancer remains unclear. We investigated the clinicopathological utility of PD-L1 expression in HER2-positive breast cancer. Cohort A included 248 patients with invasive breast cancer (all subtypes). Cohort B included 126 HER2-positive patients who received neoadjuvant chemotherapy (NAC) concomitant with trastuzumab. The relationship of PD-L1 expression on the cancer cells with clinicopathological factors including pathological complete response (pCR) and prognosis was investigated. In cohort A, 8.1% patients were PD-L1-positive; PD-L1 positivity showed a correlation with high degree of tumor-infiltrating lymphocytes (TILs), estrogen receptor negativity, progesterone receptor negativity, and high histological grade. In cohort B, 17.5% patients were PD-L1-positive; PD-L1 positivity showed a significant correlation with high degree of TILs and high abundance of CD8-positive TILs. The pCR rates were related to TILs and PD-L1 expression. Among PD-L1-negative patients, high CD8-positive TILs were associated with significantly better prognosis. In conclusion, 17.5% of HER2-positive type patients were PD-L1-positive. PD-L1 expression was associated with response to NAC with trastuzumab in patients with HER2-positive breast cancer.

## Introduction

Mononuclear immune cells located in tumor tissues [also referred to as tumor-infiltrating lymphocytes (TILs)] play an important role in tumor immunity^1,2^. The degree of TIL is considered an important prognostic factor and a predictive factor for the treatment of breast cancer patients, especially those with estrogen receptor (ER)-negative type^3–6^. In 2014, the International Working Group established guidelines pertaining to the evaluation of the degree of TIL in invasive breast cancer^7^. On the basis of these guidelines, we previously found that TIL-expression was a potent predictor of the response to neoadjuvant chemotherapy (NAC) with trastuzumab in patients with human epidermal growth factor receptor 2 (HER2)-positive breast cancer^4^.

Programmed cell death protein-1 (PD-1) and programmed death-ligand 1 (PD-L1) are considered as immune checkpoint factors that inhibit the immune reaction to cancer cells^8,9^. Thus, these factors have attracted attention as novel therapeutic targets in the context of many cancer types^10–12^. In lung cancer, immunohistochemical (IHC) assay of PD-L1 expression is the companion and/or complementary diagnostic tool for the PD-1/PD-L1 immune checkpoint inhibitors^13^. Several clinical trials have investigated the use of PD-1/PD-L1 immune checkpoint inhibitors in patients with invasive breast cancer^14–16^. In addition, the PD-L1 antibody SP142 was used as the companion diagnosis in a clinical trial that investigated the use of atezolizumab (a PD-L1 inhibitor) for treatment of triple-negative breast cancer^14^. However, the clinical utility of PD-L1 evaluation in the context of HER2-positive breast cancer is not clear.

The present study investigated the relationship of PD-L1 expression with several clinicopathological factors, including the outcome and pathological response to NAC with trastuzumab in patients with HER2-positive breast cancer.

## Results

### Clinicopathological and prognostic utility of PD-L1 expression

**Figure 1:**
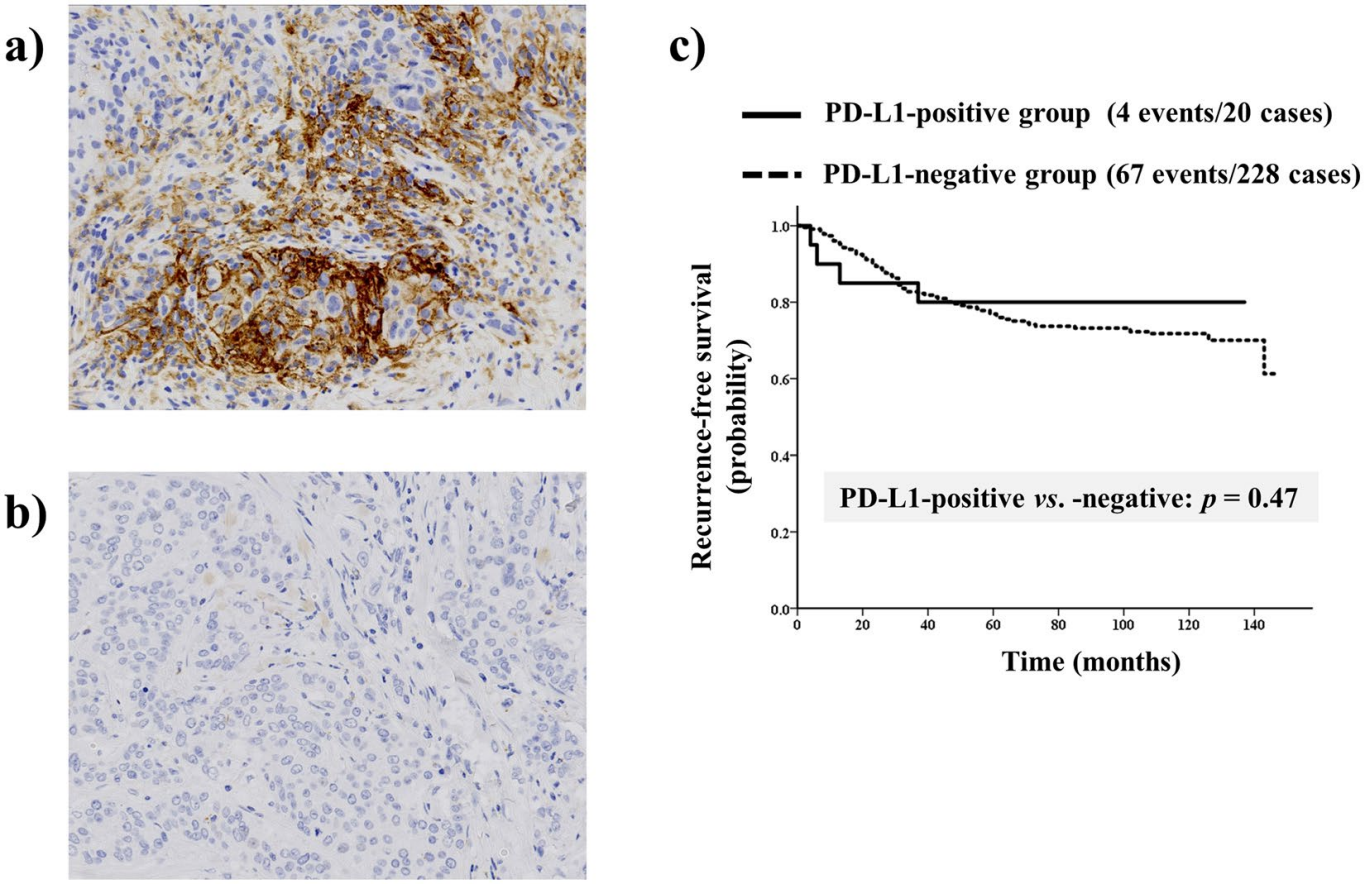
Immunohistochemical staining of programmed death-ligand 1 (PD-L1) to assay protein expression in breast cancer tissue showing (**a**) positive-staining and (**b**) negative-staining in the cytoplasm. (**c**) Cumulative survival of all subtypes of breast cancer patients stratified according to PD-L1 expression. PD-L1 was not a significant prognostic factor in any of the subtypes of breast cancer.

In cohort A (n = 248), 20 patients (8.1%) were PD-L1-positive (Fig. 1a) while 228 (91.9%) patients were PD-L1-negative (Fig. 1b). PD-L1 positivity showed a significant association with ER negative status (*p* < 0.0001), PgR negative status (*p* < 0.0001), high TIL expression (*p* < 0.0001) and histological grade 3 (*p* < 0.0001) (Table 1). In addition, 1.3% of the hormone receptor (HR)-positive/HER2-negative type, 11.6% of the HER2-positive type and 27.7% of the triple-negative type were classified as PD-L1-positive breast cancer in cohort A. The median recurrence-free survival (RFS) in cohort A was 128 (range, 1–147) months. PD-L1 expression was not a significant prognostic factor in any of the subtypes of breast cancer (hazard ratio = 0.69, 95% confidence interval (CI) 0.25–1.88, *p* = 0.47; Fig. 1c). On multivariate analyses, PD-L1 expression was not an independent prognostic factor (hazard ratio = 0.51, 95% confidence interval (CI) 0.17–1.56, *p* = 0.24; Table S1).

**Table 1:** Correlation of PD-L1 expression with clinicopathological factors in invasive breast cancer.

		PD-L1 expression		*N*	*p*
		Negative	Positive		
TILs	High	18 (58.1%)	13 (41.9%)	31	<0.0001
	Intermediate	39 (95.1%)	2 (4.9%)	41	
	Low	171 (97.2%)	5 (2.8%)	176	
ER	Negative	63 (77.8%)	18 (22.2%)	81	<0.0001
	Positive	165 (99.8%)	2 (1.2%)	167	
PgR	Negative	87 (83.7%)	17 (16.3%)	104	<0.0001
	Positive	141 (97.9%)	3 (2.1%)	144	
HER2	Positive	38 (88.4%)	5 (11.6%)	43	0.35
	Negative	190 (92.7%)	15 (7.3%)	205	
Subtypes	HR-positive and HER2-negative	156 (98.7%)	2 (1.3%)	158	<0.0001
	HER2-positive	38 (88.4%)	5 (11.6%)	43	
	Triple negative	34 (72.3%)	13 (27.7%)	47	
Histological grade	Grade 3	125 (86.2%)	20 (13.8%)	145	<0.0001
	Grade 1/2	103 (100.0%)	0 (0.0%)	103	
Pathological tumor size	pT 3/4	110 (90.9%)	11 (9.1%)	121	0.56
	pT 1/2	118 (92.9%)	9 (7.1%)	127	
Pathological nodal status	Positive	101 (91.0%)	10 (9.0%)	111	0.62
	Negative	127 (92.7%)	10 (7.3%)	137	

Abbreviations: PD-L1, programmed death-ligand 1; TILs, tumor-infiltrating lymphocytes; ER, estrogen receptor; PgR, progesterone receptor; HER2, human epidermal growth factor receptor 2; HR, hormone receptor.

### Correlation of PD-L1 expression with clinicopathological factors including pathological response to NAC with trastuzumab

In cohort B (n = 126), 22 patients (17.5%) were PD-L1-positive, while 104 patients (82.5%) were PD-L1-negative (Table 2). PD-L1 positivity showed a significant association with high expression of TIL (*p* < 0.0001), high abundance of CD8-positive TIL (*p* = 0.00087) and histological grade 3 (*p* = 0.043) (Table 1). The distribution of clinicopathological factors including PD-L1 expression in the pCR and non-pCR groups is presented in Table 3. The pCR rate showed a significant correlation with PD-L1 expression (*p* = 0.027). The pCR rate in PD-L1-positive patients (86.4%) was significantly greater than that in PD-L1-negative patients (61.5%) (Table 3). As shown in Table 3, pCR also showed a significant association with high (40–90%) TIL expression (*p* = 0.027), ER negativity (*p* < 0.0001), PgR negativity (*p* = 0.00015), high (≥30%) Ki67 labelling index (*p* = 0.0052) and histological grade 3 (*p* = 0.0038). However, on multivariate analysis, none of these factors was an independent predictor of pCR (Table 3).

**Table 2:** Correlation of PD-L1 expression with clinicopathological factors in HER2-positive breast cancer.

		PD-L1 expression		*N*	*p*
		Negative	Positive		
TILs	High	6 (26.1%)	17 (73.9%)	23	<0.0001
	Intermediate	34 (91.9%)	3 (8.1%)	37	
	Low	64 (97.0%)	2 (3.0%)	66	
CD8-positive TILs	High	65 (74.7%)	22 (25.3%)	87	0.00087
	Low	30 (100.0%)	0 (0.0%)	30	
ER	Negative	62 (80.5%)	15 (19.5%)	77	0.45
	Positive	42 (85.7%)	7 (14.3%)	49	
PgR	Negative	74 (81.3%)	17 (18.7%)	91	0.56
	Positive	30 (85.7%)	5 (14.3%)	35	
Ki67	High (≥30%)	70 (77.8%)	20 (22.2%)	90	0.36
	Low (<30%)	34 (94.4%)	2 (5.6%)	36	
Histological grade	Grade 3	85 (79.4%)	22 (20.6%)	107	0.043
	Grade 1/2	19 (100.0%)	0 (0.0%)	19	
Clinical tumor size	cT 3/4	38 (86.4%)	6 (13.6%)	44	0.41
	cT 1/2	66 (80.5%)	16 (19.5%)	82	
Clinical nodal status	Positive	68 (80.0%)	17 (20.0%)	85	0.28
	Negative	36 (87.8%)	5 (12.2%)	41	

Abbreviations: PD-L1, programmed death-ligand 1; TILs, tumor-infiltrating lymphocytes; ER, estrogen receptor; PgR, progesterone receptor HER2, human epidermal growth factor receptor 2.

**Table 3:** Relationship between pathological complete response and clinicopathological factors including PD-L1 expression.

		Non-pCR	pCR	pCR ratio (%)	*p*	
					Univariate	Multivariate
PD-L1	Positive	3	19	86.4	0.027	0.34
	Negative	40	64	61.5		
TILs	40–90%	3	20	87.0	0.018	0.40
	0–40%	40	63	61.2		
CD8-positive TILs	High	26	61	70.1	0.18	—
	Low	13	17	56.7		
ER	Negative	15	62	80.5	<0.0001	0.058
	Positive	28	21	42.9		
PgR	Negative	22	69	75.8	0.00015	0.25
	Positive	21	14	40.0		
Ki67	High (≥30%)	24	66	73.3	0.0052	0.15
	Low (<30%)	19	17	47.2		
Histological grade	Grade 3	31	76	71.0	0.0038	0.28
	Grade 1/2	12	7	36.8		
Clinical tumor size	cT 3/4	17	27	61.4	0.43	—
	cT 1/2	26	56	68.3		
Clinical nodal status	Positive	29	56	65.9	1.00	—
	Negative	14	27	65.9		

Abbreviations: PD-L1, programmed death-ligand 1; TILs, tumor-infiltrating infiltrating lymphocytes; ER, estrogen receptor; PgR, progesterone receptor; pCR, pathological complete response.

**Figure 2:**
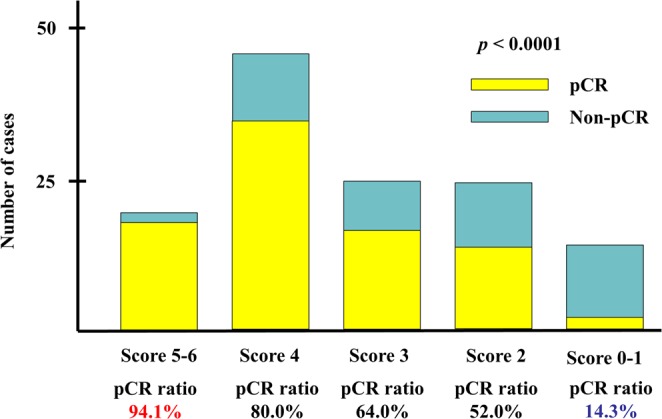
The pathological complete response (pCR) prediction scores and their relationship with pCR. The pCR score, consisting of programmed death-ligand 1 (PD-L1), tumor-infiltrating lymphocytes (TILs), estrogen receptor (ER), progesterone receptor (PgR), Ki67, and histological grade 3 significantly predicted pCR.

We assessed the relationship of pCR rate with the combination of the following 6 biological markers: PD-L1, TILs, ER, PgR, histological grade 3, and Ki67. All 126 patients were classified into 7 groups (score 0–6). The pCR rates of patients with score 5–6, score 4, score 3, score 2, and score 0–1 were 94.1%, 80.0%, 64.0%, 52.0%, and 14.3%, respectively. This scoring was a significant predictor of pCR (*p* < 0.0001) (Fig. 2).

### Prognostic utility of PD-L1 expression in HER2-positive type

**Figure 3:**
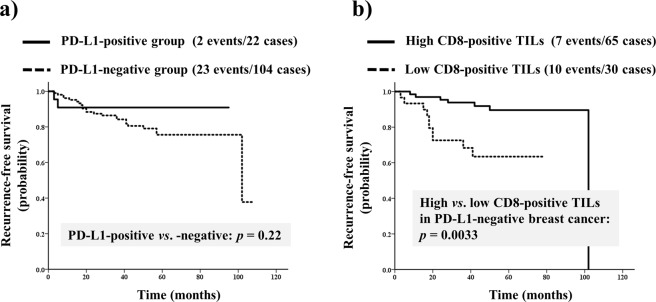
(**a**) Cumulative survival of patients with human epidermal growth factor receptor 2 (HER2)-positive breast cancer stratified according to programmed death-ligand 1 (PD-L1) expression. PD-L1 was not a significant prognostic factor in HER2-positive breast cancer. (**b**) Survival curves stratified according to the degrees of CD8-positive tumor-infiltrating lymphocytes (TILs) in PD-L1-negative/HER2-positive patients. Among PD-L1-negative/HER2-positive patients, recurrence-free survival was significantly better in the high CD8-positive TILs group than in the low CD8-positive TILs group.

The median survival in cohort B (n = 126) was 52.5 (range, 3–108) months. In this cohort of HER2-positive patients, PD-L1 expression was not a significant prognostic factor (hazard ratio = 0.40, 95% CI 0.09–1.71, *p* = 0.22; Fig. 3a). The other 5 biological markers associated with pCR (i.e. PD-L1, TILs, ER, PgR, histological grade 3, and Ki67) showed no significant association with prognosis (Table S2). However, among patients with PD-L1-negative breast cancer, survival in the high CD8-positive TIL expression group was significantly longer than that in the low CD8-positive TIL expression group (hazard ratio = 4.58, 95% CI 1.66–12.67, *p* = 0.0033; Fig. 3b).

## Discussion

PD-1 is expressed on the surface of lymphocytes, whereas its ligand PD-L1 is expressed on the surface of cancer cells as well as lymphocytes^8^. PD-1 and PD-L1 belong to the immune checkpoint family of proteins, which inactivate T-lymphocytes. Within the tumor microenvironment, PD-1/PD-L1 expression suppresses the immune response by killer/cytotoxic T cells against cancer cells^8^. This type of mechanism, mediated via modulation of PD-1 and PD-L1 binding, represents the resistance of tumor cells to anti-tumor immunity. Discovery of methods to overcome these mechanisms of tumor resistance while maintaining the initial anti-tumor immune response is a key area of immune oncology research^9^. Over the previous three decades, various PD-1/PD-L1 inhibitors have been developed for the treatment of several types of cancer^17^. Clinical trials have suggested the efficacy of PD-L1 inhibitors against solid tumors, including skin cancer^18^, lung cancer^19^, and bladder cancer^20^. In a worldwide clinical study, use of a PD-L1 inhibitor in combination with nab-paclitaxel was shown to significantly prolong the progression-free survival of patients with metastatic triple-negative breast cancer compared to nab-paclitaxel monotherapy^14^.

Several studies have shown the clinicopathological significance of PD-L1 expression and the usefulness of PD-1/PD-L1 inhibitors in the treatment of triple-negative breast cancer^16,21,22^. However, only a few clinical trials have investigated the role of PD-1/PD-L1 inhibitors in patients with HER2-positive breast cancer. Results of the PANACEA clinical trial pertaining to patients with metastatic HER2-positive breast cancer, showed a 15.2% objective response rate to pembrolizumab-trastuzumab combination therapy in PD-L1-positive patients as against 0% in PD-L1-negative patients^23^. In HER2-positive breast cancer, the anti-tumor immune response is also an important predictor of therapeutic response and prognosis. In our previous study, high expression of TILs in the primary tumor was associated with significant improvement in pCR rate after NAC with trastuzumab^4^. Trastuzumab is an anti-HER2-targeting drug, which binds and inhibits HER2 dimerization, resulting in inhibition of the downstream phosphatidylinositol 3-kinase cascade^24,25^. In addition, trastuzumab induces anti-tumor effects by promoting the activation of natural killer cells through antibody-dependent-cellular cytotoxicity (ADCC)^26,27^. In our previous study, NAC with trastuzumab was found to induce an increase in the number of TILs in 20% of non-pCR cases, compared with pre-treatment measurements. Furthermore, the group with high TILs in the residual tumor showed a significantly better prognosis than the group with low TILs^4^. Trastuzumab induces infiltration of lymphocytes in the tumor independent of the ADCC activity. Perez *et al*.^28^ conducted a study using large-scale transcriptomic data; they found a strong association between the gene sets related to immune function and the effect of trastuzumab therapy in patients with HER2-positive breast cancer. In the present study, all PD-L1-positive cases showed a high density of CD8-positive TILs. In contrast, in the PD-L1-negative cases, the positivity of CD8-positive TILs was a significant prognostic factor. Collectively, these findings suggest that trastuzumab is strongly associated with killer/cytotoxic T cell activity, in addition to ADCC activity^4^.

The effectiveness of combination therapy with immune checkpoint inhibitors and anti-HER2 agents against early-stage HER2-positive breast cancer is an important topic in medical oncology. However, further investigation is necessary to determine the most suitable patients for such combination therapy^29^. PD-L1 expression in breast cancer cells may be a predictive biomarker of response to PD-1/PD-L1 immune checkpoint inhibitors. However, there is no clear consensus on the evaluation method for PD-L1 expression with respect to the selection of the PD-L1 antibody clone and the appropriate percentage cutoff level to determine PD-L1 positivity and negativity^30^. Representative PD-L1 antibodies include 22C3, 28-8, SP263, and SP142. SP142 binds to the cytoplasmic domain of PD-L1, whereas 22C3 and 28-8 bind to its extracellular domain^31,32^. Of note, 22C3 was used in a companion diagnostic assay for non-small-cell lung cancer in a clinical trial of pembrolizumab therapy. In that study, the cutoff values were set at ≥50% and ≥1% for first- and second-line treatment, respectively^33,34^. Furthermore, 28-8 was used in a complementary diagnostic assay in a clinical trial of nivolumab therapy for non-small-cell lung cancer; in this study, the cutoff value was set at ≥1% for second-line treatment^8,35^. In the IMpassion130 trial^14^ – assessing the efficacy of atezolizumab against triple-negative breast cancer – SP142 was used with a cutoff value of ≥1%. However, in the Blueprint Working Group study, the positivity rate obtained with the use of SP142 was lower than that obtained with 22C3, 28-8, and SP263^36^. Furthermore, in a study by Sun *et al*.^37^, the PD-L1 expression patterns were found to differ depending on the antibody clone used; they reported a positivity rate of 19.3% with use of SP142. In the present study, we used the PD-L1 antibody SP142 with a cutoff value of ≥1%. On this basis, 1.3% of HR-positive/HER2-negative cases, 11.6% of HER2-positive cases, and 27.7% of triple-negative cases were classified as PD-L1-positive. Further prospective studies are warranted to identify appropriate antibodies for PD-L1 companion and complementary diagnoses in breast cancer.

IHC assessment revealed increased PD-L1 expression in cancer cells and low expression in normal epithelial cells^8^. However; PD-L1 expression on the tumor cells is heterogeneous, and PD-L1 inhibitors may bind to PD-L1-positive cancer cells as well as PD-L1-positive lymphocytes^38^. The difference between the effects of PD-L1 inhibitors on PD-L1-positive cancer cells and lymphocytes is not clear. In the current study, PD-L1 expression on tumor cells was not a prognostic biomarker in patients with HER2-positive breast cancer. In a recent study, PD-L1 expression on tumor cells was associated with high-risk clinicopathological parameters and poor prognosis, while PD-L1 expression on the TILs was associated with favourable survival outcomes. In the present study, we did not evaluate PD-L1 expression on TILs. Further studies are warranted to delineate the mechanisms that underlie PD-L1 expression in tumor cells and lymphocytes.

In conclusion, approximately 15% of HER2-positive breast cancers were found PD-L1-positive using the PD-L1 antibody SP142. PD-L1 expression on cancer cells was found to be a predictive biomarker for response to NAC with trastuzumab. A limitation of the present study is that the clinical utility of PD-L1 expression was not clear for HER2-positive breast cancer treated with immune check point inhibitors. Several studies are currently underway to identify biomarkers that may predict the response to these inhibitors; these broadly focus on the character of TILs, PD-L1 expression, the mutational landscape, and the gene signature. In this context, it is important to determine the differences in the characteristics of immune cells (e.g. CD8-positive TILs) prior to and after NAC with trastuzumab and to advance the evaluation of PD-L1 expression, with the objective to optimise the use of immune checkpoint inhibitors in the treatment of HER2-positive breast cancer.

## Methods

### Patient characteristics

Cohort A comprised of 248 consecutive female patients with invasive breast cancer who received breast cancer surgery without NAC at the Saitama Cancer Center, Saitama, Japan between 2000 and 2001 (Table S3).

Among patients with invasive breast cancer who underwent surgery at the Division of Breast Surgery in Saitama Cancer Center between 2005 and 2011, 126 consecutive patients with HER2-positive type who received NAC with trastuzumab were included in cohort B. All patients received 24 cycles of trastuzumab with NAC consisting of paclitaxel or docetaxel followed by anthracycline (Table S4). The details of the treatment administered in cohort B are described elsewhere^39^.

Ethical approval for this study was provided by the Institutional Review Board of the Saitama Cancer Center (Reference numbers: 533 and 534). Informed consent was obtained from all patients included in the study.

### Histopathological evaluation

IHC and *in-situ* hybridisation were performed as described elsewhere^39,40^. Briefly, the following antibodies were used for IHC staining to determine the subtype: ER (1D5; DAKO, Copenhagen, Denmark), progesterone receptor (PgR; PgR636; DAKO), and HER2 (HercepTest; DAKO). Specimens with a nuclear staining rate ≥1% were considered HR-positive (ER and PgR). HER2 amplification was automatically stained (BenchMark^®^ XT; Ventana Medical Systems, Tucson, Arizona, USA) with dual *in-situ* hybridisation (DISH; INFORM HER2 Dual ISH DNA Probe Cocktail Assay; Roche, Basel, Switzerland). Patients with ‘HER2 IHC-score 3+’ or ‘HER2 IHC-score 2+ and those positive for HER2 amplification by DISH’ were defined as HER2-positive type. IHC staining for Ki67 (MIB-1, DAKO) was performed automatically using an IHC machine (BenchMark^®^ XT, Ventana Medical Systems, Inc.). The Ki67 labelling index (percentage of positivity) was calculated for approximately 500 cancer cells in hot to warm areas.

Pathological complete response (pCR) to NAC was evaluated in accordance with the guidelines of the Japanese Breast Cancer Society. The details of this evaluation are described elsewhere^39^. Residual noninvasive cancers or axillary lymph node metastases were not considered while determining the pCR.

### Evaluation of biomarkers associated with tumor immunity

The surgical samples were used in cohort A, and the core needle biopsy specimens were used in cohort B.

PD-L1 expression was assessed by IHC using SP142 (dilution 1:50; Spring Biosience, USA). Breast cancer cells with a cytoplasmic and/or membrane staining rate ≥1% were classified as PD-L1-positive (Fig. 1a). Staining was assessed by an investigator specialising in breast pathology (MK) according to the evaluation method initially established for urothelial cancer^41^.

Haematoxylin and eosin-stained sections were used for the evaluation of TILs. For this purpose, the expression of mononuclear immune cells interposing between tumor nests (stromal TILs) was evaluated using an optical microscope (magnification: 200–400x); the evaluation was performed by an investigator specialised in breast pathology (MK). The expression of TIL, as previously reported^3,4^, was categorised into three groups by modifying the International Working Group criteria^7^: low (TILs: 0–10%); moderate (TILs: 10–40%); high (TILs: 40–90%). In cohort B, the expression of CD8 (DAKO, Denmark) in TILs was evaluated by IHC staining of core needle biopsy specimens of primary tumours. Presence of >25 CD8-positive TILs in one high-power field was defined as ‘high CD8-positive TIL expression’^4^.

For the assessment of the relationship between pCR rate and the combination of 6 biological markers (i.e. PD-L1, TILs, ER, PgR, histological grade 3, and Ki67), one point each was assigned for PD-L1-positivity, high TIL expression, ER-negativity, PgR-negativity, high Ki67 labelling index and histological grade 3.

### Statistical analysis

The association of PD-L1 expression with pCR rate and clinicopathological factors was assessed using the Chi-squared test. Multivariate logistic regression analysis was performed to identify factors significantly associated with pCR. In addition, the association of PD-L1 and CD8 expressions with prognosis [RFS] was determined using the Cox proportional hazards model. Survival curves were drawn using the Kaplan–Meier method. All statistical analyses were performed using the SPSS statistical software version 24.0 (IBM, Armonk, New York, USA); *p* values ≤ 0.05 were considered indicative of statistical significance.

### Ethics approval and consent to participate

This study was conducted according to the tenets of the Declaration of Helsinki. Ethic approval was granted by the Saitama Cancer Center Institutional Review Board (Reference numbers: 533 and 534). Informed consent was obtained from all patients included in the study.

## Supplementary information

Supplementary Tables

## Data Availability

The datasets generated and/or analysed during the current study are available from the corresponding author on reasonable request.
