# Supplementary material for: Clinicopathological values of PD-L1 expression in HER2-positive breast cancer
Source: Sci Rep. 2019 Nov 13;9:16662. doi: 10.1038/s41598-019-52944-6 (PMC6853939; doi:10.1038/s41598-019-52944-6)
Supplement: Supplementary file 1 — Supplementary Tables [file 41598_2019_52944_MOESM1_ESM.pdf]

Clinicopathological values of PD-L1 expression in HER2-positive breast cancer

Sasagu Kurozumi, Kenichi Inoue, Hiroshi Matsumoto, Takaaki Fujii, Jun Horiguchi, Tetsunari Oyama, Masafumi Kurosumi, and Ken Shirabe

Supplementary Table 1. Results of univariate and multivariate survival analyses showing the influence of clinicopathological factors, including PD-L1 expression, in cohort A (all subtypes of breast cancer)

		Univariate analysis			Multivariate analysis		
		Hazard ratio	95% CI	*p*-value	Hazard ratio	95% CI	*p*-value
PD-L1	Negative	Reference			Reference		
	Positive	0.69	0.25–1.88	0.47	0.51	0.17–1.56	0.24
TILs	0%–40%	Reference			Reference		
	40%–90%	1.13	0.56–2.27	0.74	0.85	0.38–1.89	0.69
ER	Positive	Reference			Reference		
	Negative	1.64	1.02–2.64	0.40	1.02	0.47–2.21	0.95
PgR	Positive	Reference			Reference		
	Negative	1.81	1.14–2.89	0.013	1.80	0.89–3.64	0.10
HER2	Negative	Reference			Reference		
	Positive	2.00	1.17–3.42	0.011	1.51	0.83–2.74	0.18
Histological grade	Grade 1/2	Reference			Reference		
	Grade 3	1.51	0.92–2.47	0.10	1.12	0.63–1.97	0.71
Pathological tumor size	pT 1/2	Reference			Reference		
	pT 3/4	2.31	1.42–3.77	0.00081	1.96	1.18–3.24	0.0090
Pathological nodal status	Negative	Reference			Reference		
	Positive	3.89	2.32–6.53	<0.0001	3.50	2.06–5.95	<0.0001

Abbreviations: PD-L1, programmed death-ligand 1; TILs, tumor infiltrating lymphocytes; ER, estrogen receptor; PgR, progesterone receptor; HER2, human epidermal growth factor receptor 2: pCR, pathological complete response; CI, confidence interval

Supplementary Table 2. Results of univariate survival analysis of the 6 factors associated with pathological complete response, including PD-L1 expression in the cohort B (HER2-positive breast cancer with neoadjuvant treatment with trastuzumab)

		Univariate analysis		
		Hazard ratio	95% CI	*p*-value
PD-L1	Negative	Reference		
	Positive	0.40	0.09–1.71	0.22
TILs	0%–40%	Reference		
	40%–90%	0.38	0.09–1.60	0.19
ER	Positive	Reference		
	Negative	0.67	0.31–1.48	0.32
PgR	Positive	Reference		
	Negative	0.60	0.27–1.33	0.21
Ki67	Low (< 30%)	Reference		
	High (≥ 30%)	0.69	0.30–1.57	0.37
Histological grade	Grade 1/2	Reference		
	Grade 3	1.11	0.37–3.27	0.86

Abbreviations: PD-L1, programmed death-ligand 1; TILs, tumor infiltrating lymphocytes; ER, estrogen receptor; PgR, progesterone receptor; HER2, human epidermal growth factor receptor 2

Supplementary Table 3. Characteristics of patients in cohort A (all subtypes of breast cancer)

Age range (years)		Pathological tumour size	
≤40	32	pT1	127
40< and <60	132	pT2	98
≥60	84	pT3	16
Menopausal status		pT4	7
Premenopausal	108	Pathological nodal status	
Postmenopausal	140	pN0	138
Type of breast surgery		pN1	62
Breast-conserving surgery	197	pN2	30
Mastectomy	51	pN3	18
Axillary surgery		Pathological TNM stage	
Sentinel lymph node biopsy alone	130	I	86
Axillary lymph node dissection	118	IIA	83
Subtypes		IIB	27
HR-positive and HER2-negative	158	IIIA	27
HER2-positive	43	IIIB	7
Triple-negative	47	IIIC	18
Abbreviations: HR, hormonal receptor; HER2, human epidermal growth factor receptor 2; TNM, TNM Classification of Malignant Tumours			

Supplementary Table 4. Characteristics of patients in cohort B (HER2-positive breast cancer treated with neoadjuvant therapy with trastuzumab)

Age range (years)		Clinical tumour size	
≤40	20	cT1	6
40< and <60	74	cT2	80
≥60	32	cT3	25
Menopausal status		cT4	15
Premenopausal	50	Clinical nodal status	
Postmenopausal	76	cN0	41
Type of breast surgery		cN1	57
Breast-conserving surgery	107	cN2	17
Mastectomy	19	cN3	11
Axillary surgery		Clinical TNM stage	
Sentinel lymph node biopsy alone	53	I	2
Axillary lymph node dissection	73	IIA	37
		IIB	37
		IIIA	28
		IIIB	11
		IIIC	11

Abbreviations: HER2, human epidermal growth factor receptor 2; TNM, TNM Classification of Malignant Tumours
